# Presentations, Complications, and Challenges Encountered During Management of Type 1 Diabetes in Egyptian Children During COVID-19 Pandemic: A Single-Center Experience

**DOI:** 10.3389/fendo.2022.814991

**Published:** 2022-03-11

**Authors:** Marise Abdou, Mona M. Hassan, Samah A. Hassanein, Eman H. Elsebaie, Radwa A. Shamma

**Affiliations:** ^1^ The Diabetes, Endocrine and Metabolism Pediatric Unit, Pediatric Department, Cairo University, Cairo, Egypt; ^2^ Public Health and Community Medicine Department, Cairo University, Cairo, Egypt

**Keywords:** presentations, complications, challenges, type 1 diabetes, COVID-19, children and adolescents

## Abstract

**Background:**

The coronavirus disease 2019 (COVID-19) pandemic has been associated with significant challenges pertaining to the management of children and adolescents with type 1 diabetes (T1D). Issues such as fear of infection and lockdown measures have resulted in delayed and more severe clinical presentations of this disease.

**Objectives:**

This study aimed at reporting the frequency and severity of diabetic ketoacidosis (DKA) and the rate of DKA complications in children with diabetes who presented to the emergency unit during COVID-19 pandemic. Furthermore, the purpose of this study was to compare the data collected from the first and second COVID-19 waves with that of the pre-COVID-19 period and describe the challenges encountered during disease management.

**Methods:**

This cross-sectional study included all children and adolescents with T1D who presented to the emergency department at Abo El Rish Children’s Hospital, Cairo University, during the first and second COVID-19 waves. It also included data collected from the pre-COVID-19 period. Demographic and clinical data, investigations, and management details were collected from the patients’ medical records.

**Results:**

Three hundred twenty-four Egyptian children and adolescents diagnosed with T1D were recruited. One hundred forty patients (43.2%) presented with severe DKA, and approximately 66% were newly diagnosed with T1D. The participants presented with manifestations suggestive of COVID-19, such as fever (29.5%), respiratory manifestations (7.2%), and gastrointestinal symptoms (14.7%). Thirty-seven patients were tested for severe acute respiratory syndrome coronavirus 2 infection using nasopharyngeal swabs, and four patients tested positive. Around 18% of patients developed hypokalemia during disease management. A comparison between these data and the data from previous years revealed that there was a significant increase in the number of newly diagnosed cases with more severe DKA at presentation and a higher frequency of development of hypokalemia during both COVID-19 waves.

**Conclusion:**

An increase in the frequency of newly diagnosed cases was identified during the first and the second COVID-19 waves compared with the pre-COVID-19 period. The patients presented with more severe DKA, probably due to a more delayed presentation. The frequency of hypokalemia development was also significantly higher, and the severity of DKA was associated with a longer ICU admission. Further studies are required to establish a definitive link between the COVID-19 pandemic and the severity of presentation.

## Introduction

Coronavirus disease 2019 (COVID-19) is an infectious respiratory syndrome caused by severe acute respiratory syndrome coronavirus 2 (SARS-CoV-2) (1). Among the various modes of COVID-19 virus transmission, droplet infection is the most common, which is increased by close contact with positive cases (2, 3). Furthermore, it has been suggested that this virus can be transmitted through the fecal–oral route, as it was found to be shed in stools and urine. It is also found in the tears of infected individuals (2, 3). The clinical presentations of a COVID-19 infection can vary from asymptomatic to severely infected cases. It can sometimes lead to death (2). The symptoms in infected children are more likely to be mild. According to the findings of a Chinese study on children who tested positive, typical symptoms were found in 45% of cases, mild respiratory symptoms were found in 42%, 13% were asymptomatic, and no children had life-threatening symptoms (4). Children with immunocompromising conditions, such as diabetes, may be at a higher risk of a more severe disease (5).

The COVID-19 pandemic poses significant challenges to the management of children and adolescents with type 1 diabetes (T1D), especially in newly diagnosed cases. These challenges have affected both the institutional and self-management levels, particularly in limited-resource settings (6). More specifically, this pandemic has changed the way that patients access healthcare settings—from scheduled clinic and hospital visits to telemedicine (7).

Parents were reluctant in seeking prompt medical advice and therefore delayed the presentation of children with diabetes. Multiple factors contributed to this, including the fear of spread of infection especially among these vulnerable patients, the overwhelmed medical system by the care of SARS-CoV-2-infected patients in addition to the limited resources, and the lockdown measures. This might have affected the outcome especially in cases presenting with diabetic ketoacidosis (DKA) (8). Additionally, the need of managing children and adolescents with T1D in the emergency unit had affected the duration until the resolution of DKA and imposed an additional risk of infection. They were required to stay in the emergency unit along with other patients until all necessary investigations to exclude a COVID-19 infection were completed, in accordance with our center’s policy. Provided that they test negative, these patients were transferred to the inpatient ward at the Diabetes, Endocrine, and Metabolism Pediatric Unit (DEMPU).

Even after DKA resolution, the length of hospital stay had to be minimized to reduce the risk of infection during admission. After discharge, children with diabetes were followed up *via* phone as an alternative way to regularly scheduled clinic visits in an attempt to minimize the risk of exposure to infection. This inadequate approach induced an additional load over both physicians and parents in limited-resource settings that lack the availability of continuous glucose monitoring (CGM). This was particularly noted in children who were recently diagnosed with diabetes and were still devastated by the diagnosis (9).

Data on the development, clinical presentation, and management of T1DM (both new onset and known) during the SARS-CoV-2 pandemic in Egyptian children are limited, with no available comparisons with patients during the pre-COVID-19 period. Therefore, the aims of this study were to identify the challenges during management and follow-up of children and adolescents with T1D and to report the severity and frequency of DKA together with the rate of DKA complications. Finally, we aimed at comparing the collected data with data from the pre-COVID-19 period.

## Materials and Methods

This cross-sectional study included Egyptian children and adolescents with T1D who presented to the emergency department at the Abo El Rish Children’s Hospital, Cairo University, between June and July 2020 (first COVID-19 wave) and between December 2020 and February 2021 (second COVID-19 wave). All patients who presented with DKA or hyperglycemia either as a first presentation or with known diabetes were included. Their ages varied from 6 months up to 18 years. DKA was confirmed by the classic triad of hyperglycemia (blood glucose >200 using a glucometer), metabolic acidosis (pH <7.3, HCO_3_ <15), and ketonuria using urinary dipsticks (10).

The patients were recruited at presentation to the ER during the first and the second COVID-19 waves after obtaining verbal consent from the patients and/or their parents and legal guardians. Data from the pre-COVID-19 period was collected retrospectively by reviewing the patients’ medical records. The DEMPU records were reviewed, and the following data were collected:

- Age, gender, onset of diabetes (newly diagnosed or known T1D), initial presentation (DKA or hyperglycemia), and the presence of signs of a neurological affliction.
- Duration of ICU admission (if applicable) and the full length of hospital stay.
- Symptoms suggestive of a COVID-19 infection.
- Management details especially pertaining to the antishock therapy received and the development of hypokalemia that necessitated correction.
- Investigations performed at presentation, including random blood glucose, ketone bodies in urine (using urinary dipsticks), and venous blood gases, upon which patients were classified into mild, moderate, and severe DKA (mild DKA: pH, <7.3; HCO_3_, <15; moderate DKA: pH, < 7.2, HCO_3_, <10; severe DKA: pH, < 7.1; HCO_3_, <5) (10). Furthermore, electrolytes (Na and K), renal functions (urea and creatinine), and complete blood count (CBC) with differential (especially total lymphocyte counts) and C-reactive protein (CRP) were also included. Serum K levels <3.5 mEq/L were also used to diagnose hypokalemia. Hypokalemia was graded into mild (serum K, 3–3.4 mEq/L), moderate (serum K, 2.5–2.9 mEq/L), and severe (serum K, <2.5 mEq/L) (11).
- Radiological data, including computed tomography (CT) of the chest and brain in selected cases presenting with neurological symptoms and signs, were included.

The initial screening for a COVID-19 infection, according to hospital policy, included CBC and CT chest examinations. If CT was suspicious of COVID-19 and/or CBC revealed lymphopenia and/or leukopenia, the patients were kept in an isolation unit until a test swab was done to exclude or confirm a COVID-19 diagnosis. The normal lymphocyte count in adults is 1,000–4,800/µl (1–4.8 × 10^9^/L; in children <2 years, the normal count is 3,000–9,500/µl (3–9.5 × 10^9^/L). At the age of 6 years, the lower limit of normal count is 1,500/µl (1.5 × 10^9^/L). Different laboratories may have slightly different normal values (12).

## Results

A total of 324 Egyptian children and adolescents with T1D were included in this study. One hundred fifteen patients were recruited during the first COVID-19 wave and 209 patients during the second COVID-19 wave. Male individuals constituted 51.2% of all patients, and the female individuals were at 48.8%. The participants’ age ranged from 6 months to 18 years. Regarding the initial presentation during the first and the second COVID-19 waves, 70 patients (21.6%) presented with hyperglycemia, and 254 patients (78.4%) presented with DKA. One hundred forty patients (43.2%) had severe DKA, and 45% of them were admitted during the first COVID-19 wave and 55% during the second COVID-19 wave.

During both COVID-19 waves, 213 participants (65.7%) were newly diagnosed with T1D, and 111 (34.3%) were known to have diabetes. In total, 23% of the newly diagnosed patients presented with hyperglycemia, 9% with mild DKA, 17.3% with moderate DKA, and 50.7% with severe DKA. Whereas, 19% of patients known to have diabetes presented with hyperglycemia, 18% with mild DKA, which is approximately double the cases of newly diagnosed diabetes, 34.2% with moderate DKA, which is higher than the newly diagnosed group, and 28.8% with severe DKA. A comparison of the demographic and clinical data of the studied groups is presented in **Table 1**.

**Table 1:** Comparison between the first and the second COVID-19 waves regarding the patients’ demographic, clinical, laboratory, and radiological data.

Variables studied	First wave		Second wave		*P*-value
	Total (115)		Total (209)		
	*N*	%	*N*	%	
Gender					0.629
Male Female	61	53.0%	105	50.2%	
	54	47.0%	104	49.8%	
Diabetes status					0.118
First presentation Known to have T1D	82	71.3%	131	62.7%	
	33	28.7%	78	37.3%	
Positive clinical manifestations suggestive of COVID-19 infection	57	49.6%	33	15.9%	<0.001^a^
Fever	53	93.0%	25	12.1%	<0.001^a^
Respiratory symptoms suggestive of infection	6	10.5%	13	6.3%	0.272
Gastrointestinal symptoms suggestive of infection	8	13.8%	31	15.0%	0.822
Neurological manifestations (low GCS and/or seizures)	5	8.8%	28	13.5%	0.336
Metabolic status at presentation					0.011^a^
Hyperglycemia Mild DKA Moderate DKA Severe DKA	22	19.1%	48	23.0%	
	8	7.0%	31	14.8%	
	22	19.1%	53	25.4%	
	63	54.8%	77	36.8%	
Total leukocytic count at presentation					1.000
Leukocytosis Leukopenia Normal	53	46.5%	80	41.2%	
	0	0.0%	7	3.6%	
	61	53.5%	107	55.2%	
Lymphocytic count at presentation					0.315
Lymphocytosis Lymphopenia Normal	7	6.1%	7	3.6%	
	19	16.7%	24	12.4%	
	88	77.2%	162	83.9%	
CRP at presentation					<0.001^a^
Positive Negative Not done	28	24.3%	17	8.2%	
	61	53.0%	42	20.3%	
	26	22.6%	148	71.5%	
CT chest suspicious of COVID-19 infection	14	12.5%	12	5.8%	0.037^a^
Nasopharyngeal swab for COVID-19					0.108
Positive Negative Not done	3	2.6%	1	0.5%	
	15	13.0%	18	8.7%	
	97	84.4%	189	90.9%	
Complications during DKA management					<0.001^a^
Hypokalemia No hypokalemia Hypernatremia Other	5 110	4.3% 95.7%	54 154	26.0% 74%	
	1	0.9%	4	1.9%	
	10	8.7%	6	2.9%	

a P-value less than 0.05 is considered statistically significant.

N, number; CT, computed tomography; CRP, C-reactive protein; GCS, Glasgow Coma Scale; T1D, Type 1 diabetes.

The mean age and SD of patients during the first and the second COVID-19 waves were 7.96 ± 3.55 and 8.1 ± 3.57 years, respectively (*p* = 0.722), revealing no statistically significant difference between both groups regarding age. The frequency of severe DKA was noted to be significantly higher during the first COVID-19 wave (54.8% of the cases) compared to the second wave (36.8% of the cases). Additionally, it was noted that the median bicarbonate level of patients admitted during the first wave was 4 mEq/L (range, 0.8–47 mEq/L), which was statistically significantly lower than the median bicarbonate level of patients admitted during the second wave (7.65 mEq/L, ranging from 0.8 to 27.9 mEq/L) (*p* = 0.003). There were statistically significant differences between the first and the second COVID-19 waves regarding the presence of features suggestive of a COVID-19 infection, such as fever, severity of initial presentation, presence of suspicious CT chest findings, and positive CRP (*p* < 0.001, *p* < 0.001, *p* = 0.011, *p* < 0.001, and *p* < 0.037 respectively). **Table 1** shows that these features were more prevalent during the first COVID-19 wave.

**Figure 1:**
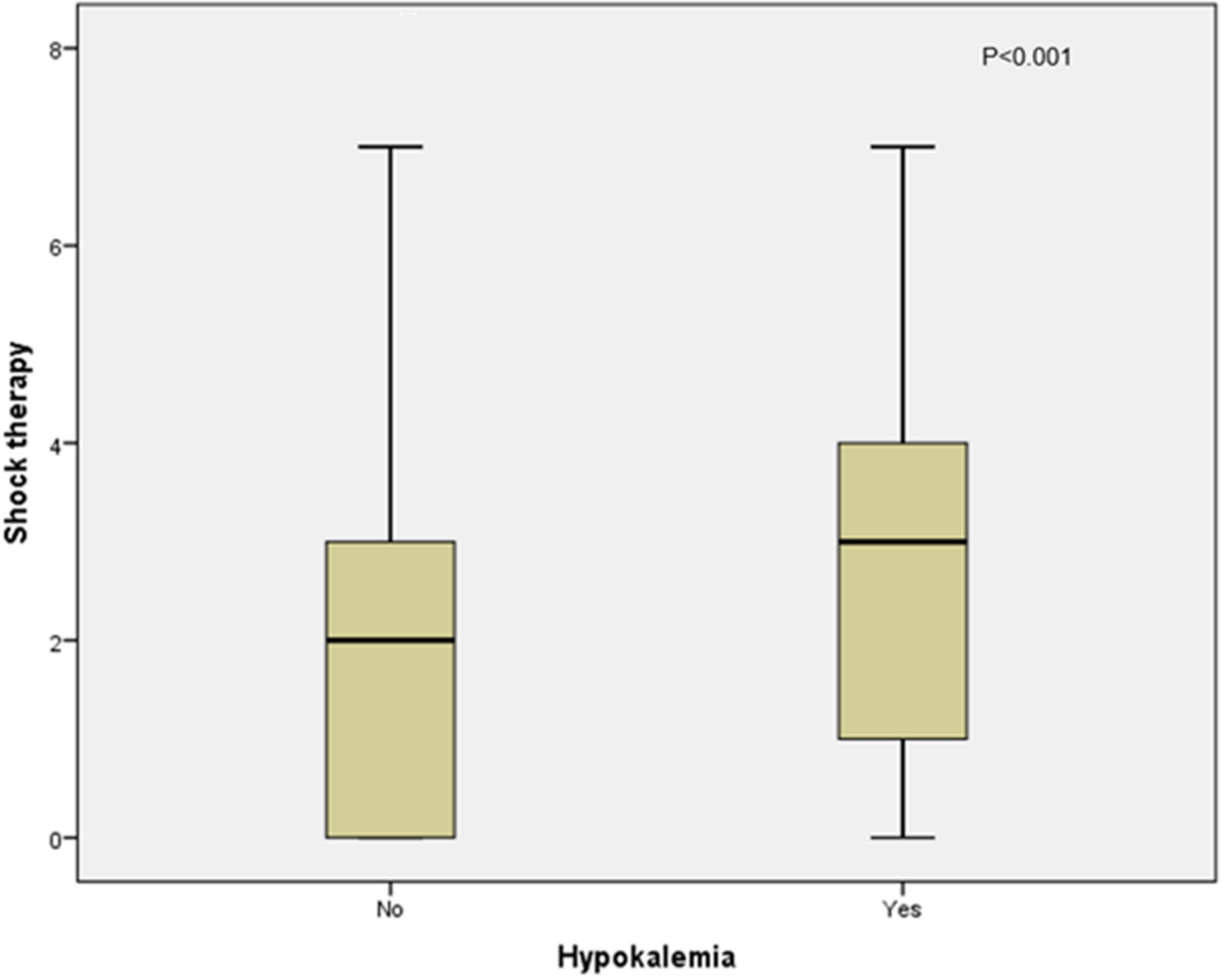
Association between hypokalemia and the amount of shock therapy received in the second COVID-19 wave.

**Figure 2:**
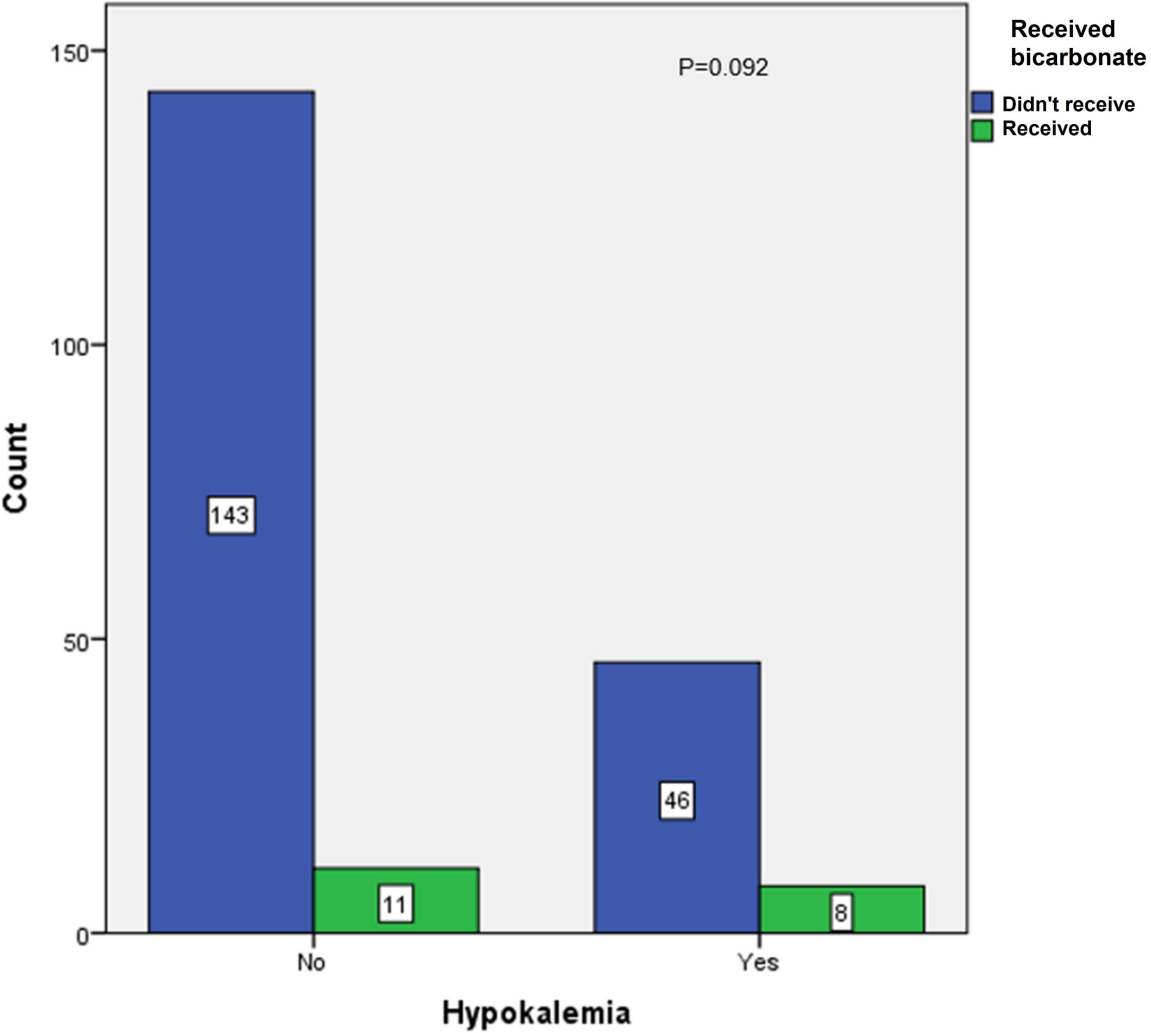
Association between hypokalemia and intake of sodium bicarbonate in the second COVID-19 wave.

**Figure 3:**
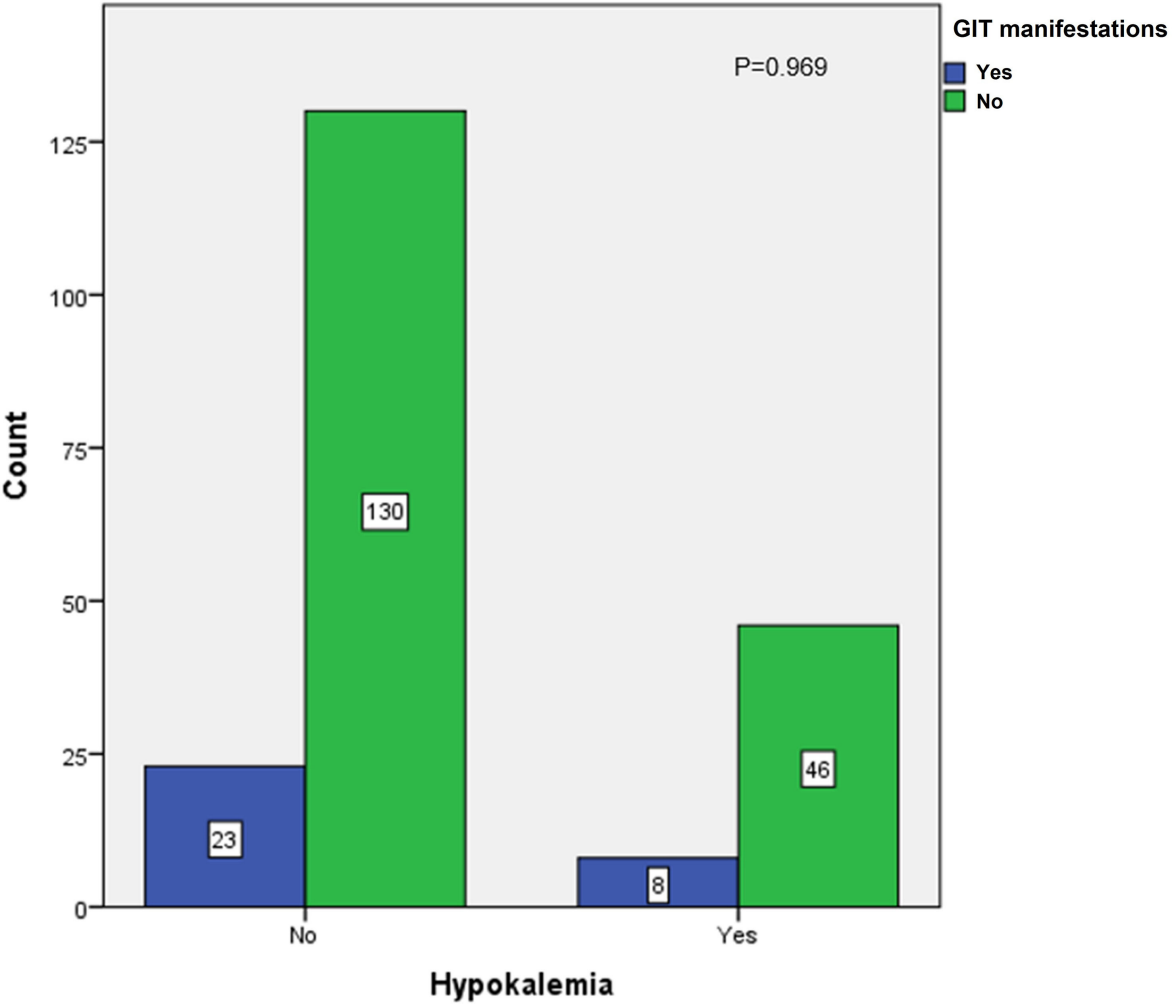
Association between hypokalemia and development of gastrointestinal manifestations in second COVID-19 wave.

In the present study, 14 patients (7.4%) admitted during the second COVID-19 wave had hypokalemia at presentation—eight of them presented with mild hypokalemia, four had moderate hypokalemia, and two had severe hypokalemia—whereas the number of patients who developed hypokalemia as a complication during management in both COVID waves was 59, making up 18.3% of the study group (5 patients during the first COVID-19 wave and 54 patients during the second COVID-19 wave). Out of the 54 who developed hypokalemia as a complication during the second COVID-19 wave, 26 (12.5%) received oral correction and 28 patients (13.5%) received intravenous correction. A statistically significant association was observed between the rates of hypokalemia that developed during management and the amount of shock therapy received (*p* < 0.001), as shown in **Figure 1**. In contrast, there was no statistically significant association between hypokalemia development and intravenous bicarbonate treatment in some cases of severe DKA (*p* = 0.092) (**Figure 2**) or with the development of gastrointestinal symptoms (*p* = 0.969) in admitted patients with diabetes (**Figure 3**).

### First COVID-19 Wave

The mean age of patients who presented with severe DKA was 7.6 ± 3.63 years (mean pH of 6.93 ± 0.105 and mean HCO_3_ of 2.707 ± 1.018 mEq/L). In total, 58.7% of patients who presented with severe DKA were female individuals, and 41.3% were male individuals (*p* < 0.021, statistically significant). Approximately 77.8% of patients with severe DKA were newly diagnosed with T1D, whereas 22.2% were patients with known diabetes (*p* = 0.014, statistically significant), as shown in **Table 2**.

**Table 2:** Association of the grades of severity of DKA and the enlisted variables in the first COVID-19 wave.

	Initial presentation								*P*-value
	Hyperglycemia		Mild DKA		Moderate DKA		Severe DKA		
	*N*	%	*N*	%	*N*	%	*N*	%	
Gender									0.021^a^
Male Female	17	77.3%	4	50.0%	14	63.6%	26	41.3%	
	5	22.7%	4	50.0%	8	36.4%	37	58.7%
Diabetes status									0.014^a^
First presentation Known to have T1D	17	77.3%	2	25.0%	14	63.6%	49	77.8%	
	5	22.7%	6	75.0%	8	36.4%	14	22.2%
Fever	4	80.0%	4	80.0%	7	100.0%	38	95.0%	0.339
Gastrointestinal symptoms suggestive of infection	1	20.0%	1	20.0%	1	12.5%	5	12.5%	0.942
Respiratory symptoms suggestive of infection	0	0.0%	0	0.0%	0	0.0%	6	15.0%	0.415
Neurological manifestations (low GCS and/or seizures)	2	40.0%	1	20.0%	0	0.0%	2	5.0%	0.041^a^
CT chest suspicious of COVID-19 infection	5	23.8%	1	12.5%	0	0.0%	8	12.9%	0.141
Total leukocytic count at presentation									<0.001
Leukocytosis Leukopenia Normal	2	9.5%	3	37.5%	5	22.7%	43	68.3%	
	0	0.0%	0	0.0%	0	0.0%	0	0.0%
	19	90.5%	5	62.5%	17	77.3%	20	31.7%
Lymphocytic count at presentation									0.232
Lymphocytosis Lymphopenia Normal	2	9.5%	0	0.0%	0	0.0%	5	7.9%	
	2	9.5%	2	25.0%	1	4.5%	14	22.2%
	17	81.0%	6	75.0%	21	95.5%	44	69.8%
CRP at presentation									0.157
Positive Negative Not done	5	22.7%	4	50.0%	3	13.6%	16	25.4%	
	9	40.9%	4	50.0%	12	54.5%	36	57.1%
	8	36.4%	0	0.0%	7	31.8%	11	17.5%
Nasopharyngeal swab for COVID-19									0.452
Positive Negative Not done	1	4.5%	0	0.0%	0	0.0%	2	3.2%	
	4	18.2%	1	12.5%	0	0.0%	10	15.9%
	17	77.3%	7	87.5%	22	100.0%	51	81.0%
Complication during DKA management									0.093
Hypokalemia Hypernatremia Other complications	0	0.0%	0	0.0%	4	18.2%	1	1.6%	
	0	0.0%	0	0.0%	0	0.0%	1	1.6%
	3	13.6%	0	0.0%	0	0.0%	7	11.1%

a P-value less than 0.05 is considered statistically significant.

N, number; TLC, total leukocytic count; CRP, C-reactive protein; CT, computed tomography; DKA, diabetic ketoacidosis; GCS, Glasgow Coma Scale; T1D, Type 1 diabetes.

Only few patients (5%) who presented with severe DKA suffered from neurological symptoms (low Glasgow Coma Scale score and/or seizures) (*p* = 0.041, statistically significant). However, a high proportion of these patients showed leukocytosis with a longer duration of stay in the ICU (*p* < 0.001 for both). There were no other statistically significant associations with other variables, as shown in **Table 2**.

### Second COVID-19 Wave

The mean age of patients admitted with severe DKA during this wave was 7.8 ± 3.48 years. There was no predilection to a certain gender among this group. The mean blood glucose of patients with severe DKA was 585 ± 175.27 mg/dl, with a mean pH value of 6.91 ± 0.103, mean HCO_3_ value of 3.1 ± 1.23 mEq/L, and mean serum creatinine value of 0.976 ± 0.363 mg/dl. There were statistically significant differences in these parameters when compared with the values obtained from patients admitted with lesser grades of DKA or with hyperglycemia (*p* < 0.001). The mean total leukocytic count was statistically higher among subjects with severe DKA (25.39 × 10^3^, *p* < 0.001). The mean time spent in the ICU (33 h) was also significantly longer (*p* < 0.001). Additionally, the mean number of times that patients with severe DKA received shock therapy was 3.92 times (each was 10 cc/kg), which was significantly more than those with milder presentations (mild DKA, moderate DKA, or hyperglycemia) (*p* < 0.001). Fifty-nine patients (76.6%) with severe DKA were newly diagnosed with T1D, whereas 18 patients (23.4%) were known to have T1D (*p* = 0.001).

Furthermore, we observed that the increased severity of DKA was associated with a significantly higher total leukocytic count and higher frequency of developing complications, especially hypokalemia, during management (*p* < 0.001 for both). Despite the high number of cases presenting with severe DKA, only few suffered from neurological manifestations (in the form of low Glasgow Coma Scale score and seizures) (*p* < 0.001). During DKA management, only 19 out of 77 (24.7%) patients received IV bicarbonate therapy (*p* < 0.001). Finally, we did not identify any statistically significant association with other variables, as shown in **Table 3**.

**Table 3:** Association of the grades of severity of DKA and the enlisted variables in the second COVID-19 wave.

	Initial presentation								*P*-value
	Hyperglycemia		Mild DKA		Moderate DKA		Severe DKA		
	*N*	%	*N*	%	*N*	%	*N*	%	
Gender									0.266
Male Female	22	45.8%	17	54.8%	32	60.4%	34	44.2%	
	26	54.2%	14	45.2%	21	39.6%	43	55.8%
Diabetes status									0.001^a^
First presentation Known to have T1D	32	66.7%	17	54.8%	23	43.4%	59	76.6%	
	16	33.3%	14	45.2%	30	56.6%	18	23.4%
Fever	3	6.5%	4	12.9%	4	7.5%	14	18.2%	0.166
Gastrointestinal symptoms suggestive of infection	3	6.5%	5	16.1%	7	13.2%	16	20.8%	0.189
Respiratory symptoms suggestive of infection	3	6.5%	2	6.5%	0	0.0%	8	10.4%	0.124
Neurological manifestations (low GCS and/or seizures)	1	2.2%	2	6.5%	2	3.8%	23	29.9%	<0.001^a^
CT chest suspicious of COVID-19 infection	3	6.5%	3	9.7%	1	1.9%	5	6.5%	0.484
Total leukocytic count at presentation									<0.001^a^
Leukocytosis Leukopenia Normal	1	2.3%	8	26.7%	17	33.3%	54	77.1%	
	4	9.3%	1	3.3%	2	3.9%	0	0.0%
	38	88.4%	21	70.0%	32	62.7%	16	22.9%
Lymphocytic count at presentation									0.680
Lymphocytosis Lymphopenia Normal	1	2.3%	0	0.0%	2	4.0%	4	5.7%	
	5	11.6%	2	6.7%	8	16.0%	9	12.9%
	37	86.0%	28	93.3%	40	80.0%	57	81.4%
CRP at presentation									0.026^a^
Positive Negative Not done	2	4.3%	2	6.5%	0	0.0%	13	17.1%	
	10	21.3%	7	22.6%	10	18.9%	15	19.7%
	35	74.5%	22	71.0%	43	81.1%	48	63.2%
Nasopharyngeal swab for COVID-19									0.933
Positive Negative Not done	0	0.0%	0	0.0%	0	0.0%	1	1.3%	
	4	8.5%	3	9.7%	5	9.4%	6	7.8%
	43	91.5%	28	90.3%	48	90.6%	70	90.9%
Received bicarbonate	0	0.0%	0	0.0%	0	0.0%	19	24.7%	<0.001^a^
Inotropic support	1	2.1%	0	0.0%	0	0.0%	5	6.5%	0.104
Cause of DKA									0.445
Not known Missed doses Infection	5	50.0%	0	0.0%	7	31.8%	3	25.0%	
	4	40.0%	6	85.7%	14	63.6%	8	66.7%
	1	10.0%	1	14.3%	1	4.5%	1	8.3%
Hypokalemia	5	10.6%	6	19.4%	8	15.1%	35	45.5%	<0.001^a^
Hypernatremia	0	0.0%	1	3.2%	0	0.0%	5	6.5%	0.087
Admission in isolation	4	8.3%	3	9.7%	7	13.2%	7	9.1%	0.844

a P-value less than 0.05 is considered statistically significant.

N, number; TLC, total leukocytic count; CRP, C-reactive protein; CT, computed tomography; DKA, diabetic ketoacidosis; GCS, Glasgow Coma Scale; T1D, Type 1 diabetes.

A confirmation of diagnosis of SARS-CoV-2 infection in suspected cases was done by performing COVID-19 PCR using nasopharyngeal swabs. This procedure was performed in 37 children who fulfilled the local testing criteria (clinical, laboratory, and imaging). Only four of them tested positive (three in the first COVID-19 wave and one in the second COVID-19 wave). The patients had to stay for 48 h in the emergency department waiting for the result of the nasopharyngeal swab. This was associated with a significantly longer ICU stay (mean duration of ICU stay was 45.5 h for whom a swab was done *versus* 24.5 h for whom a swab was not done, *p* = 0.002), However, this long stay in the ER was not associated with more complications (*p* = 0.581).

### Comparison Between the First and the Second COVID-19 Waves and the Pre-COVID-19 Period

This study also compared the data of patients with T1D admitted during the COVID-19 pandemic and of patients admitted during the pre-COVID-19 period. During the pre-COVID-19 period, 60 patients were admitted to the Abo El Rish Hospital between January and February 2018. Twenty-three of them (38.3%) were newly diagnosed diabetes cases, whereas 61.7% were known to have diabetes. Those numbers were significantly different from the number of patients admitted during the COVID-19 pandemic (where 65.7% of patients were newly diagnosed and 34.3% were known diabetic patients, *p* < 0.001) (**Table 4**).

**Table 4:** Comparison of data between the COVID-19 waves and pre-COVID.

Laboratory data at presentation	COVID waves *N* (319)	Pre-COVID *N* (60)	*P*-value
	Mean ± SD	Mean ± SD	
Initial pH	7.1127 ± 0.20522	7.0122 ± 0.68802	0.266
Initial bicarb (mEq/L)	8.9006 ± 7.39415	11.8067 ± 8.13244	0.012^a^
Na (mEq/L)	134.5397 ± 6.59422	135.7117 ± 6.92245	0.237
K (mEq/L)	4.3555 ± 0.70945	4.5770 ± 0.92326	0.092
Serum creatinine (mg/dl)	0.8809 ± 0.31444	0.8983 ± 1.24839	0.915
Blood glucose (mg/dl)	506.1053 ± 166.17674	498.6167 ± 132.84400	0.749
Presence of ketones in urine	2.409 ± 0.9228	2.867 ± 0.3428	<0.001^a^

a P-value less than 0.05 is considered statistically significant.

During both the first and the second COVID-19 waves, we found that the mean HCO_3_ level was 8.9 mEq/L, which was significantly lower than the mean HCO_3_ level during the pre-COVID-19 period (11.8 mEq/L) (*p* = 0.012), as shown in **Table 4**. This indicates that the severity of DKA was significantly higher during the COVID-19 pandemic compared to the pre-COVID-19 period. We also noticed that hypokalemia, as a complication of DKA management, was more frequently observed during the COVID-19 pandemic compared to the pre-COVID-19 period. More specifically, 18.3% of patients admitted during the COVID-19 pandemic developed hypokalemia as a complication in comparison to 6.7% of patients in the pre-COVID-19, which was statistically significant (*p* = 0.001).

## Discussion

The current cross-sectional study was carried out to describe the characteristics of children and adolescents with T1D who presented to our center during the first and the second COVID-19 waves. Furthermore, the purpose of this study was to identify the challenges encountered during patient management and follow-up, with a focus on data pertaining to the clinical presentation and management of T1D (both new onset and known patients) during the SARS-CoV-2 pandemic, especially considering that data on children are still limited in Egypt.

During the first and the second waves of the COVID-19 pandemic, 254 (78.3%) patients presented with DKA to the emergency department at the Abo El Rish Children Hospital, 140 of whom (43.2%) had severe DKA (mean pH: 6.93 ± 0.1 SD and mean HCO_3_: 2.7 ± 1 SD). Sixty-three (45%) patients with severe DKA presented during the first COVID-19 wave and 77 (55%) patients during the second COVID-19 wave. The newly diagnosed cases presenting with severe DKA during both waves were 108 (49 patients (77.8%) during the first wave and 59 patients (76.6%) during the second wave). This finding is consistent with the observations of a UK-based research study conducted during the first wave of the SARS-CoV-2 pandemic. Children and adolescents with diabetes, aged <17 years, were included in this study. They noticed that 70% of the patients with newly diagnosed T1D and who presented with DKA had severe DKA (13). The high proportion of children presenting with severe DKA could be explained by many factors, including lockdown measures, limited resources, and the overwhelmed medical system by the care of SARS-CoV-2-infected patients, which forced many parents to be worried and reluctant to seek prompt medical advice, therefore delaying the clinical presentation. Our observations agreed with those of the research conducted to study the specific considerations in the management of patients with diabetes during the coronavirus disease pandemic (8). Additionally, the period selected for conducting the current study and patient recruitment was between December 2020 and February 2021 (second COVID-19 wave), which coincided with the seasonal increase in the frequency of T1D and in cases presenting with DKA at first presentation. Regarding the severity of DKA in patients with known diabetes, 14 (22.2%) presented with severe DKA during the first COVID-19 wave and 18 (23.4%) during the second COVID-19 wave. This might reflect the fact that this pandemic had changed the way patients access healthcare. Telemedicine had greatly replaced scheduled clinic and hospital visits, especially during the first wave where we had expected higher rates of DKA. The transition period was associated with an increased burden for both patients and physicians, especially in Egypt where we experience limited resources and lack of CGM availability. This finding was consistent with the study conducted by Ludvigsson et al. who explored the effect of the COVID-19 pandemic on the treatment of T1D in children. He found that this period represented an increased burden on both patients and physicians (9). The pandemic has had a great emotional impact and psychological stress that might have influenced the rates of new-onset disease by changing the risk of developing autoimmunity (14). Multiple factors have also affected patients living with diabetes during both waves of the pandemic, including psychological health issues, decreased physical activity, dietary changes that led to poor nutrition and weight gain, and, finally, sleep disruptions. All of those might have altered diabetes management behaviors (15–17). Moreover, individuals who were worried about COVID-19 and diabetes could have been more vulnerable for diabetes distress, acute and chronic hyperglycemia, and onset or exacerbation of depression and anxiety (2).

During both COVID-19 waves, some of the recruited patients presented with COVID-like manifestations, including fever (29.5%), respiratory manifestations in the form of rhinorrhea, pharyngitis, cough, and respiratory distress (7.2%), and gastrointestinal manifestations in the form of vomiting, abdominal pain, and diarrhea (14.7%). In accordance with our center’s policy, these patients had to remain in the emergency department in full isolation until all necessary investigations were completed to exclude a COVID-19 infection. This was considered a challenge for us, as the patients spent a significant amount of time in the emergency department, a process that might have contributed to the delayed DKA resolution. Additionally, patients were then encountering other patients presenting with various conditions, which could have increased the risk of spreading infection and subsequent development of complications during patient management, such as electrolyte disturbance and neurological complications.

A confirmation of diagnosis in patients with suspected SARS-CoV-2 infection was done by performing COVID-19 PCR using nasopharyngeal swabs. This procedure was performed in 37 children who fulfilled the local testing criteria (clinical, laboratory, and imaging). Only four of them tested positive (three in the first COVID-19 wave and one in the second COVID-19 wave). This finding was similar to the results of a study performed in the UK where the authors performed nasopharyngeal swabs in 21 children who fulfilled the local testing criteria, of whom only two tested positive (13). Children who tested positive completed their treatment in the isolation unit until DKA was resolved, received COVID-19 treatment according to the Egyptian Ministry of Health protocol, and were then discharged following improvement, while patients who tested negative for COVID-19 were transferred to the DEMPU inpatient unit to complete their therapeutic management planning while attending diabetes education sessions. Physicians and dieticians played a particularly important role in diabetes education by attempting to simplify and explain each item and process, especially to newly diagnosed cases with T1D. Phone calls were used as an alternative way to regularly schedule clinic visits to minimize the risk of exposure to infection.

In the current study, 59 patients (18.3%) developed hypokalemia as a complication during DKA management. Fifty-four (26%) of them were admitted during the second COVID-19 wave (12.5% received oral correction and 13.5% received intravenous correction). Hypokalemia was not associated with any cardiac complications, and its association with gastrointestinal manifestations was not statistically significant. This was different from what was observed in the previously mentioned UK-based study where the authors identified three patients with severe DKA who developed refractory hypokalemia. One child with SARS-CoV-2 PCR suffered from hypokalemia related to cardiac arrest but subsequently recovered following 1 day of ventilation (13).

Different factors associated with the severity of DKA were investigated during the current study. It was noted that only 5% of the severe cases during the first COVID-19 wave and 29.9% of the severe cases during the second COVID-19 wave developed neurological manifestations in the form of low Glasgow Coma Scale score and/or development of seizures (*p* = 0.041 and <0.001, respectively). This was despite the fact that lower bicarbonate levels induce a greater risk of developing neurological complications. This was similar to the study conducted by Bialo et al. who investigated the rare complications of pediatric diabetic ketoacidosis, including neurological complications (18). Additionally, we found that there was a statistically significant association between DKA severity and the presence of leukocytosis (*p* < 0.001 for both waves) and also with a longer duration of ICU stay (*p* < 0.001 for both waves).

Leukocytosis is a sign of infection, which is by far the commonest precipitating factor for DKA. Several researchers have identified a number of factors that might contribute to the occurrence of leukocytosis during DKA, especially in its most severe presentation. These include insulin deficiency, inflammatory processes, increased levels of stress hormones, such as adrenaline and cortisol, and infection. Furthermore, a significant association was detected between the total leukocytic count and the blood pH at the onset of presentation with DKA. The lower the pH value, the higher the leukocytic count (leukemoid response) (19).

A comparison between the data collected during the COVID-19 period and the pre-COVID-19 period underlines that the number of newly diagnosed cases admitted during the first and the second COVID-19 waves was significantly higher compared to that of the pre-COVID-19 period (65.7 *vs*. 38.3%, *p* < 0.001). This comes in agreement with the findings of a multicenter study conducted in the UK that observed an increase of 80% in newly diagnosed cases over a typical year before COVID-19 (13). Furthermore, another study performed in Finland showed a significant increase in the admissions of new-onset T1D cases during the COVID-19 pandemic (20). This might be explained by the high expression of angiotensin-converting enzyme 2 (ACE2) receptors on the B cells of the islets of Langerhans, which has also been proposed to be the binding site for SARS-CoV-2 (13). However, our results showed that only four patients had a positive nasopharyngeal swab for the COVID-19 virus, which makes this link doubtful, and thus further studies are required to verify this point. It was also observed that the severity of DKA was significantly higher during the COVID-19 era, a finding that might be due to the significant delay in seeking medical advice from professionals due to lockdown, overwhelmed medical system, and fear of infection (*p* = 0.012). This was also stated in the summary of the recommendations of International Society of Pediatric and Adolescent Diabetes (ISPAD) regarding children with diabetes and COVID-19 (21). The frequency of developing hypokalemia was also observed to be higher during the COVID-19 period compared to the pre-COVID period.

Intravenous infusion of insulin remains the treatment of choice for DKA management, and it should be provided in an ICU setting according to the 2018 ISPAD guidelines. However, these guidelines have been updated during the COVID-19 pandemic to help reduce the burden on the ICU services and teams who are overwhelmed by the number of critically ill COVID-19-infected patients. The new recommendations state that, in case of uncomplicated, mild, and moderate DKA, management could be carried out outside the ICU using subcutaneous insulin (22).

It has been thought that the severe acute respiratory syndrome coronavirus 2, which is the causative organism of the COVID-19 pandemic, exerts its effects on various systems, such as the respiratory system, the vascular system, and the gastrointestinal tract, including the intestines and pancreas, by binding to ACE2, a surface receptor found on various body organs. This, in turn, results to an increase in the inflammatory processes and vascular permeability of various organs, including the pancreas, which might be a direct cause for developing T1D. The rapidly spreading virus and the emergence of various mutations and strains are considered to be a red flag that warns scientists globally regarding future long- and short-term health consequences. Given the limited information on the mode of action of SARS-CoV-2 and its different effects on organs, it is hypothesized that the increasing incidence of T1D during the COVID-19 pandemic might be triggered by infections, which raise concerns about a future increase in the number of T1D cases (23).

Further studies are required to detect and describe the association between a SARS-CoV-2 infection and the incidence of new-onset diabetes, the severity of presentation, and the development of complications during management, especially hypokalemia. Furthermore, it is essential to evaluate the impact of using telemedicine as an alternative to regularly scheduled clinic visits on the long-term glycemic control of patients and on the development of both short- and long-term complications (2).

## Conclusion

A significant increase in the frequency of newly diagnosed T1D cases was evident during the first and the second COVID-19 waves compared to the pre-COVID-19 period. The patients also presented with more severe DKA, probably due to a more delayed presentation, and the frequency of hypokalemia was significantly higher. The severity of DKA was associated with a longer ICU admission. Furthermore, few patients who presented with DKA developed neurological complications. The development of hypokalemia was not associated with any cardiac consequences. The severity of DKA was associated with a longer ICU admission. Further studies are required to establish a definitive link between the COVID-19 pandemic and the severity of presentation.

## Data Availability Statement

The raw data supporting the conclusions of this article will be made available by the authors without undue reservation.

## Ethics Statement

The studies involving human participants were reviewed and approved by the bioethical research committee, Faculty of Medicine, Kasr Alainy University Hospitals, Cairo, Egypt. Written informed consent from the participants’ legal guardian/next of kin was not required for them to participate in this study in accordance with the national legislation and institutional requirements.

## Author Contributions

MA shared in study design, data collection and interpretation, critical revision of the article, drafting the manuscript, and final approval for publishing. MH shared in study design, critical revision of the article, data interpretation, and final approval for publishing. SH shared in study design, analysis and interpretation of data, critical revision of the article, and final approval for publishing. EE shared in statistical analysis and interpretation of data, article revision, and final approval for publishing. RS shared in design of study, critical revision of the article, data interpretation, drafting the manuscript, and final approval for publishing. All authors analyzed the data, discussed the results, commented on the manuscript, and finally approved it for publishing.

## Funding

This research did not receive any specific grant from funding agencies in the public, commercial, or not-for-profit sectors.

## Conflict of Interest

The authors declare that the research was conducted in the absence of any commercial or financial relationships that could be construed as a potential conflict of interest.

## Publisher’s Note

All claims expressed in this article are solely those of the authors and do not necessarily represent those of their affiliated organizations, or those of the publisher, the editors and the reviewers. Any product that may be evaluated in this article, or claim that may be made by its manufacturer, is not guaranteed or endorsed by the publisher.
